# A Retrospective Study of Rabbit Anti-T Lymphocyte Globulin (rATLG) as a Second-Line Therapy in Transplant-Ineligible Severe Aplastic Anemia Patients

**DOI:** 10.7759/cureus.98014

**Published:** 2025-11-28

**Authors:** Aritra Saha, Shailendra P Verma, Swasti Sinha, Gaurav Datta, P Raghuveer, Akshay Middinti, Rajkumar Maurya, Alpika Shukla

**Affiliations:** 1 Hematology and Oncology/Clinical Hematology, King George's Medical University, Lucknow, IND; 2 Clinical Hematology, King George's Medical University, Lucknow, IND

**Keywords:** rabbit anti-t lymphocyte globulin, second anti-t lymphocyte globulin, second-line therapy, severe aplastic anemia, very severe aplastic anemia

## Abstract

Background: Severe aplastic anemia (SAA) is a potentially fatal disorder of bone marrow failure with a high risk of life-threatening infections and bleeding. While matched sibling donor transplantation remains the preferred therapy, immunosuppressive treatment with horse anti-thymocyte globulin (hATG), cyclosporine, and eltrombopag is the standard first-line option for non-transplant candidates. However, a significant proportion of patients fail to respond, and access to allogeneic transplantation is severely restricted in resource-limited settings due to prohibitive costs and infrastructure constraints. In such scenarios, rabbit anti-T lymphocyte globulin (rATLG) emerges as an important second-line salvage therapy.

Methods: This hospital-based retrospective study included 10 patients with SAA or very severe aplastic anemia (VSAA), aged ≥13 years, treated at King George’s Medical University, Lucknow, between 2020 and 2024. All patients had relapsed or refractory disease following hATG and were ineligible for transplantation. rATLG was administered at 3.5 mg/kg/day for five days alongside cyclosporine and eltrombopag. Responses were assessed at three, six, and 12 months, with survival analysed using Kaplan-Meier (KM) estimates (censoring applied for patients alive or lost to follow-up at the last contact).

Results: The cohort had a median age of 23 years, with 70% (7/10 patients) classified as SAA and 30% (3/10 patients) as VSAA. Four had refractory disease, six had relapsed disease, and three harboured a PNH clone. At 12 months, 40% (4/10 patients) achieved partial response; no complete responses were observed. Overall survival (OS) at two years was 78% (95% CI: 40.3-94.9) by KM estimate. The most common adverse events were fever, pruritus, hepatic dysfunction, and infections.

Conclusion: Although modest in efficacy, rATLG offers a clinically meaningful salvage option in transplant-ineligible SAA patients, particularly in low-resource settings where curative transplantation is often inaccessible. Our findings underscore the role of rATLG as a bridge therapy, while highlighting the urgent need for wider access to definitive curative options in developing countries.

## Introduction

Aplastic anemia (AA) is a life-threatening hematological disorder characterized by reduced bone marrow cellularity and impaired hematopoiesis, resulting in symptoms such as fatigue, recurrent infections, and bleeding tendencies. The condition is often complicated by febrile neutropenia and hemorrhage at critical sites, including the central nervous system, thereby increasing the risk of mortality. Although decreased hematopoiesis typically affects all three blood cell lineages, the initial stages may disproportionately impact one or two lineages, eventually progressing to trilineage hypoplasia [1].

Paul Ehrlich described the first case of AA in 1885 [2]. Historically, AA was considered a fatal disease; however, with the advent of newer therapies, the outcome has significantly improved. Hematopoietic stem cell transplantation (HSCT) with a matched sibling donor (MSD) remains the primary treatment modality in patients younger than 40 years of age. In older individuals, immunosuppressive therapy is considered the first-line treatment. This typically includes horse anti-thymocyte globulin (hATG), cyclosporine, and, more recently, eltrombopag [3].

While this combination has improved treatment outcomes, with a six-month overall response rate of approximately 68% [4], a significant proportion of patients remain unresponsive. For non-responders, HSCT becomes the next line of treatment. However, in developing nations such as India, the cost of allogeneic HSCT, ranging from ₹11 to ₹52 lakhs [5], poses a substantial barrier for most patients.

The British Society for Haematology (BSH) guidelines highlight the role of a second course of ATG, including rabbit anti-T lymphocyte globulin (rATLG) as a second-line treatment in patients who have failed initial immunosuppressive therapy (IST). However, its use remains limited due to concerns about complications and modest efficacy [3].

This study was conducted to assess the response to rATLG in transplant-ineligible patients with severe aplastic anemia (SAA) who either relapsed or failed to respond to the initial hATG therapy.

## Materials and methods

The aim of this hospital-based, observational, retrospective study was to assess the treatment response to rATLG in transplant-ineligible patients with SAA who did not respond to hATG. The specific objectives were to evaluate the efficacy of rATLG as a second-line therapy at three, six, and 12 months, and to assess its side effect profile.

This study was conducted in the Department of Clinical Hematology at King George’s Medical University, Lucknow, after clearance was taken from the Institutional Ethical Committee (IEC: XXX-PGTSC-IIA/P41) and included patients treated between 2020 and 2024. The study population comprised individuals aged 13 years or older diagnosed with SAA or very SAA (VSAA), who were either unresponsive to initial hATG therapy or had lost response and were not eligible for HSCT. SAA was defined by bone marrow cellularity less than 30% (excluding lymphocytes) with at least two of the following: absolute neutrophil count <500/μL, platelet count <20,000/μL, or absolute reticulocyte count <60,000/μL. VSAA was defined by an absolute neutrophil count <200/μL with the same additional criteria as SAA.

Inclusion criteria required patients to have SAA or VSAA, who had failed previous therapy with hATG and had relapsed or were refractory to the same. Relapse was defined as the need for the reinstitution of immunosuppressive therapy following an initial response to h-ATG along with cyclosporine and with or without thrombopoietin receptor agonist; whereas, refractory disease was defined as failure to achieve a response even after six months of hATG therapy [6]. Exclusion criteria included inherited bone marrow failure syndromes, clonal hematologic disorders on cytogenetics, liver enzymes (AST or ALT) >3× ULN, serum creatinine, total bilirubin, or alkaline phosphatase >1.5× ULN, liver cirrhosis, uncontrolled infections, HIV, hepatitis B surface antigen, or hepatitis C virus positivity (unless HCV-RNA negative), moribund status, severe comorbidities, or pregnancy.

The primary objective of this study was to evaluate the efficacy of rATLG as a second-line therapy at three, six, and 12 months. Secondary objectives included the analysis of OS and the assessment of the treatment's side effect profile.

Treatment involved administration of rATLG (Grafalon®) at a dose of 3.5 mg/kg/day for five consecutive days, following premedication with methylprednisolone, acetaminophen, and diphenhydramine. Patients were hospitalized during rATLG administration and discharged once clinically stable, typically by around day 10. Serum sickness prophylaxis with oral prednisone at 1 mg/kg/day was initiated after ATLG and continued for 14 days, followed by a taper over one week. Concurrent therapy with cyclosporine and eltrombopag was maintained, and antiviral prophylaxis with acyclovir was continued for six months post-ATLG. 

A complete response was defined by hemoglobin > 10g/dL, absolute neutrophil count > 1 x10^9^ /L, and platelet count >100 x10^9^/L [7]. A partial response was defined as transfusion independence, not fulfillment of the criteria for SAA. No response was defined as patients who were still fulfilling the criteria for SAA [7].

Statistical analysis was performed using IBM SPSS Statistics for Windows, version 20.0 (released 2011, IBM Corp., Armonk, NY) and Microsoft Excel 2016 (Microsoft Corp., Redmond, WA). Continuous variables were first tested for normality using the Shapiro-Wilk test. Normally distributed data were expressed as mean ± standard deviation (SD), whereas non-normally distributed data were summarized as median (range). Categorical variables were presented as frequencies and percentages (n, %). Survival analysis was performed using the Kaplan-Meier (KM) method, with patients censored at the time of last follow-up or at 24 months if alive. Two patients lost to follow-up were censored at their last documented contact. The two-year OS estimate was presented with a 95% confidence interval (CI). Given the limited sample size, inferential statistical testing and correlation analyses were considered exploratory and interpreted descriptively, acknowledging limited statistical power.

## Results

This study was conducted in the Department of Clinical Hematology at King George’s Medical University, Lucknow, and included a total of 10 patients in a case series. All participants were male, with a median age of 23.0 ± 10.3 years. Among them, seven patients were diagnosed with severe aplastic anemia, while three had very severe disease. Four patients presented with refractory disease, and six had relapsed disease. The baseline demographic and clinical characteristics of the study cohort are summarized in Table 1.

**Table 1:** Summary of baseline characteristics hATG: horse anti-thymocyte globulin, rATLG: rabbit anti-T lymphocyte globulin

Parameters	Value
Number of patients	10
Gender-male	10
Median age (years)	23.0 ± 10.3
Disease severity	
Severe	7 (70%)
Very severe	3 (30%)
Treatment indication	
Refractory	4 (40%)
Relapsed	6 (60%)
PNH clone status	
Detected	3 (30%)
Not detected	7 (70%)
Median time between hATG and rATLG (months)	44.9 (Range: 12–83)

At the end of two years, two patients had died, and two patients were lost to follow-up. One of these patients passed away within three months of therapy and was classified as a non-responder. A detectable paroxysmal nocturnal hemoglobinuria (PNH) clone was identified in three patients. The median interval between the administration of hATG and rATLG was 44.9 months (12-83).

Response rates at the third, sixth, and 12th month post rATLG

Response to rATLG improved over time, increasing from 20% at three months to 40% at 12 months. The low initial response rate suggests a delayed therapeutic onset, while most responses occurred between six and 12 months. In our study, none of the patients achieved a complete response; all observed responses were classified as partial responses. Response rates to rATLG therapy at three, six, and 12 months are detailed in Table 2.

**Table 2:** Response rates following rATLG rATLG: rabbit anti-T lymphocyte globulin

Timepoint	Responders	Total patients	Response rate (%)
3 months	2	10	20%
6 months	3	10	30 %
12 months	4	10	40%

The distribution of disease severity, PNH clone, nature of the disease, and median interval between two ATG, according to response at 12 months, was analysed.

A small cohort of patients restricted a significant subgroup analysis; however, at the 12th month, we found that there was a better trend of response in patients with PNH clone and severe disease instead of very severe disease. Subgroup analysis of response rates at 12 months, stratified by disease severity, PNH clone status, and disease nature, is shown in Table 3.

**Table 3:** Subgroup analysis with respect to the response rate at the 12th month

Parameters		Response at the 12th month	%
Severity	Severe	3/7	42.9
	Very severe	1/3	33.3
PNH clone	Present	2/3	66.7
	Absent	2/7	28.6
Nature	Relapsed	2/6	33.3
	Refractory	2/4	50

Adverse effects

Following ATLG therapy, the most frequently reported side effect was fever, occurring in all patients (100%). Both itching (pruritus) and liver dysfunction, characterized by elevated liver enzymes (AST/ALT > 3ULN) or bilirubin (total bilirubin > 1.5 ULN), were observed in seven (70%) cases.

Metabolic and immunosuppressive complications, such as steroid-induced hyperglycemia and febrile neutropenia, were noted in four patients each (40%). Less common adverse events included diarrhea and pneumonia observed in two cases each (20%). Serious but infrequent complications included single instances of intracranial bleeding and posterior reversible encephalopathy syndrome (PRES) (1%).

This pattern of adverse effects is consistent with the known toxicity profile of ATG, encompassing infusion-related reactions, immune suppression, and rare but severe events. The frequency and percentage of adverse events following rATLG therapy are presented in Table 4.

**Table 4:** Adverse event analysis PRES: posterior reversible encephalopathy syndrome

Sr. No.	Adverse effects	(n = 10)	%
1	Fever	10	100%
2	Pruritus	7	70%
2	Hepatic dysfunction (AST/ALT > 3 UNL and/or total bilirubin >3 mg/dL)	7	70%
3	Steroid-induced hyperglycemia	4	40%
4	Febrile neutropenia	4	40%
5	Diarrhoea	2	2%
6	Pneumonia	2	2%
7	Intracranial bleed	1	1 %
8	PRES	1	1%

OS at two years

**Figure 1:**
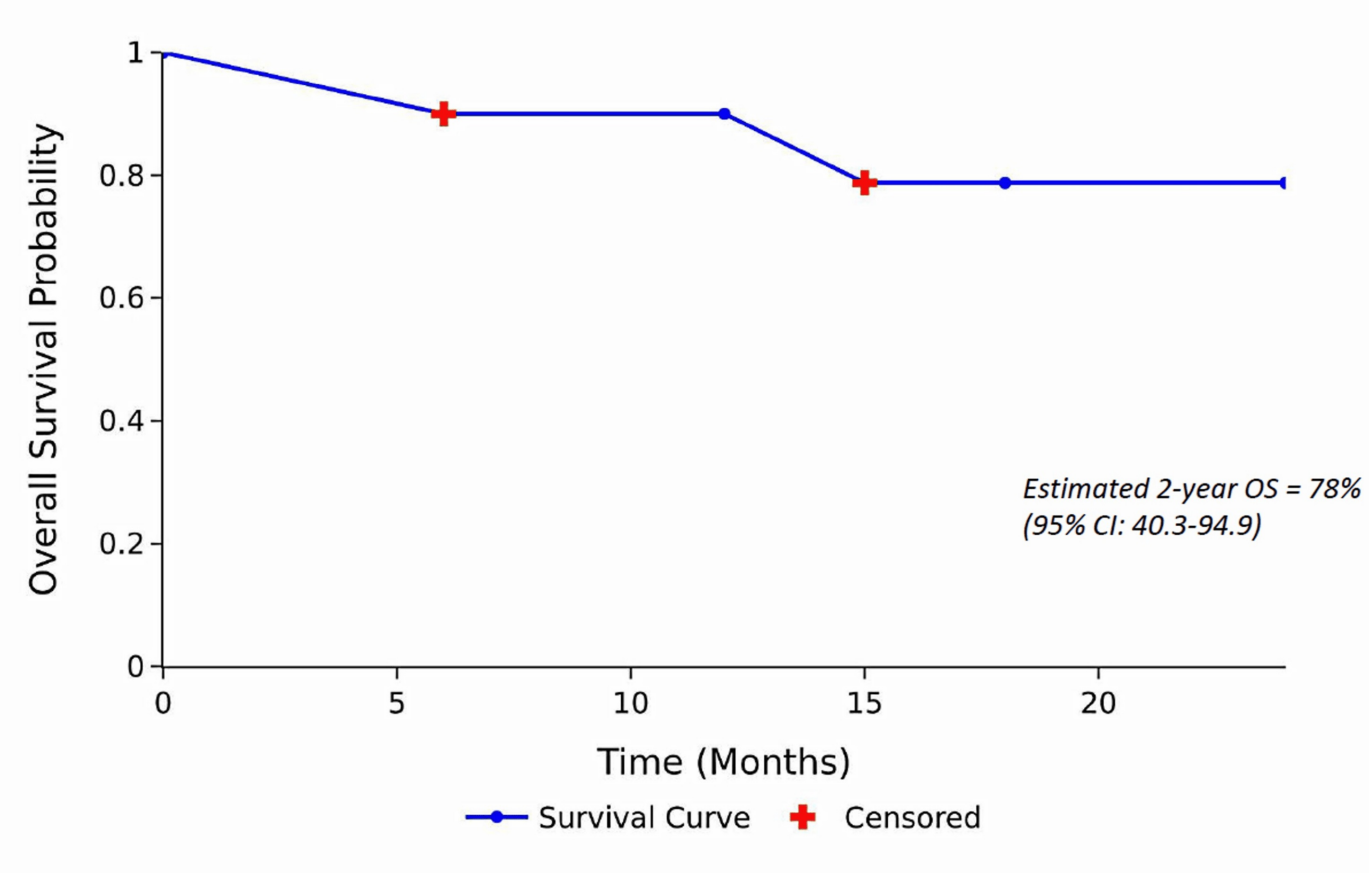
Overall survival (OS) at two years. Blue line with markers → survival curve. Red crosses → death

According to KM analysis, the estimated two-year OS was 78% (95% CI: 40.3-94.9). Two patients were lost to follow-up and were censored at their last known contact date, while two deaths were recorded during the study period. Patients with severe disease demonstrated a trend toward better survival compared to those with very severe disease, although the difference was not statistically significant due to the small cohort size. OS at two years post-rATLG treatment, estimated by KM analysis, is illustrated in Figure 1.

## Discussion

AA is an immune-mediated disorder characterized by progressive bone marrow failure, leading to considerable morbidity and mortality. Allogeneic HSCT is the definitive treatment for younger, medically fit individuals. Conversely, for older patients, the preferred therapeutic approach involves a combination of hATG, cyclosporine, and, more recently, eltrombopag. Although this regimen has significantly advanced the management of AA, it presents inherent limitations. Treatment response typically manifests four to six months after initiation of hATG, with an overall response rate ranging from 50% to 70% at the six-month mark [3].

rATLG has seen widespread use in renal transplantation and has even received FDA approval for this indication [8]. The drug works by inducing extensive T-cell depletion, a critically crucial step preceding transplantation. In earlier times, when the pathophysiology of AA was not well understood, managing such cases effectively posed a considerable challenge. Nevertheless, an improved understanding of the disease subsequently opened avenues for novel treatment approaches. Immunosuppressive therapy was established as the standard of care once it became clear that T-cell-mediated autoimmunity plays a pivotal role in the etiology of bone marrow failure [9]. Given the presumed immune-mediated destruction of stem cells as the primary pathological mechanism in AA, it was hypothesized that rATLG might be a superior agent to hATG due to its known ability for more profound lymphodepletion. The seminal study by Scheinberg et al., however, fundamentally altered this view, demonstrating that the use of hATG was associated with markedly superior response rates (68% vs. 37% at six months) [6]. Consequently, hATG has since become the universally recognized cornerstone of immunosuppressive therapy for AA. The role of rATLG has been limited to its use as a second-line therapy after failure of hATG or in conditioning regimens prior to allogenic bone marrow transplant [10].

An earlier study by Scheinberg et al. investigated the role of rATLG in the relapsed/refractory setting for transplant-ineligible patients. The investigators found that for refractory cases, the overall response rates at three and six months were 26% and 33%, respectively. By contrast, for relapsed patients, the overall response rates at three and six months were 55% and 68%, respectively. In the same study, the OS at day 1000 was 70%, with a better trend in relapsed cases than refractory cases, although it was not statistically significant [11].

Better results were seen in an Italian study involving 30 patients who had not responded to a first course of h-ALG plus cyclosporin and granulocyte colony-stimulating factor (G-CSF). These patients were given a second course using rATLG. Overall response, defined as transfusion independence, was achieved in 23 out of 30 (77%) patients, with a complete response rate of 30%. The researchers reported an astounding OS of 93% after a median follow-up of 914 days [12].

A more recent, smaller study from Japan examined outcomes in relapsed/refractory AA patients who had previously received rATLG-based immunosuppressive therapy instead of hATG. This study included 19 patients, and the overall response rates at three, six, and 12 months were 53%, 58%, and 58%, respectively. At four years, the investigators reported an OS of 100% in their cohort, and a failure-free survival (FFS) was 47.4% [13].

This study, conducted on a relatively small cohort of SAA patients, faced limitations in interpreting outcomes concerning disease severity and the nature of the disease (relapsed or refractory). Despite this, the overall response rates showed a gradual improvement over time. As previously noted, the overall response rates (ORR) at three, six, and 12 months were 20%, 33.3%, and 40%, respectively.

The two-year OS in our study was 78%, despite a poor socioeconomic background.

A potential contributing factor to the superior outcomes seen in other studies is the interval between hATG and rATLG immunosuppressive therapy (IST). In our study, the median interval between the two ISTs was highly variable, averaging 44.9 months. This is in stark contrast to studies such as Di Bona et al., which reported a median interval of only 151 days (range: 58-361 days). Similarly, Scheinberg et al. noted a median gap of 205 days (57-2,024 days) for refractory cases and 770 days (414-3,968 days) for relapsed cases. These comparisons suggest that earlier initiation of a second IST in patients unresponsive to initial therapy might lead to improved results.

In our study, fever was the most common adverse reaction, observed in nearly all patients. Pruritus was also often reported alongside fever during rATLG administration. Hepatic dysfunction, defined as AST/ALT levels exceeding three times the upper limit of normal and/or serum total bilirubin greater than 3 mg/dL, was a common finding in our patients, a contrast to observations in another study. Other adverse effects, including hyperglycemia, febrile neutropenia, and pneumonia, were comparable to those reported in previous studies [13,14].

Owing to this exceptionally small sample size, we were severely restricted in our ability to conduct robust subgroup analyses. Consequently, it proved impossible to calculate meaningful correlations between key clinical factors, such as disease severity, the specific type of the disease (whether relapsed or refractory), and the presence of a paroxysmal nocturnal hemoglobinuria (PNH) clone, and patient outcomes. However, our findings align with what has been seen with other studies, such as better response rates in patients with severe disease rather than very severe disease and in patients with PNH clone. Surprisingly, a better response rate was also observed in patients with refractory disease rather than relapsed disease, which is probably due to a relatively higher number of relapsed patients in an already small group of study population.

The most significant constraint of our research was the markedly limited study population, which served as a major obstacle to a comprehensive analysis. Furthermore, the diminutive scale of our cohort resulted in a pronounced lack of heterogeneity among the participants, which inherently limits the generalizability and statistical power of our findings. Beyond the inherent challenges of sample size, a large external factor influenced the study's execution: the socioeconomic status of our patient population. All enrolled patients faced significant financial constraints, rendering them heavily reliant on public or charitable schemes to gain free access to ATG therapy. This dependence on external funding mechanisms often introduces considerable treatment delays. As a direct consequence, adhering to a uniform, standardised treatment protocol for all patients became a formidable challenge, leading to unavoidable variations in care that could affect overall treatment efficacy and study results. It is important to acknowledge that this study was conducted in a resource-constrained setting, where logistical and financial limitations significantly influence treatment decisions. Such constraints inevitably affect treatment outcomes and OS.

Considering the limitations of our study, we acknowledge that the relatively small sample size has reduced the statistical power, thereby affecting the generalizability of our findings. To the best of our knowledge, this study represents one of the first of its kind conducted in a developing country, and as such, it may be regarded as a pilot investigation warranting further validation through larger-scale studies.

In the context of contemporary therapeutic advances, notably in HSCT, the utility of rATLG has become increasingly limited and may be perceived as outdated. However, a substantial proportion of patients in developing countries, such as India, lack access to transplantation due to financial and infrastructural constraints, leaving them with few viable treatment alternatives. While rATLG does not constitute a breakthrough intervention, it offers demonstrable, albeit modest, therapeutic benefit in this setting. 

## Conclusions

This study demonstrates that rATLG confers a modest yet clinically meaningful benefit in patients with severe or very severe aplastic anemia who are ineligible for hematopoietic stem cell transplantation and have experienced failure with initial hATG therapy. Although response rates improved over time, all observed responses were partial, and early mortality continued to be a significant challenge. These findings emphasize the critical importance of early identification of non-responders to facilitate timely therapeutic escalation. While rATLG remains a viable salvage option, its limited efficacy underscores the pressing need to expand access to curative interventions and to develop optimized treatment protocols tailored to low-resource settings. Furthermore, larger studies are necessary to validate and expand upon the results of this analysis.
